# The MRI Spectrum of Gynecological Pelvic Masses

**DOI:** 10.7759/cureus.106177

**Published:** 2026-03-31

**Authors:** Shipra Chaudhary, Ashish K Shukla, Bhoomi Modi, Manisha Gupta, Sachi Mall, Ranjeet Singh

**Affiliations:** 1 Radiodiagnosis, Santosh Medical College, Santosh Deemed to be University, Ghaziabad, IND; 2 Obstetrics and Gynecology, Santosh Medical College, Santosh Deemed to be University, Ghaziabad, IND

**Keywords:** adenomyosis, dermoid cysts, endometrial neoplasms, leiomyoma, magnetic resonance imaging, ovarian cysts

## Abstract

Background: Gynecological pelvic masses comprise a wide spectrum of benign and malignant conditions. Accurate preoperative characterization is crucial for optimal management. Although ultrasonography is the initial imaging modality, magnetic resonance imaging (MRI) provides superior soft-tissue characterization and multiplanar assessment.

Objective: To evaluate the role of MRI in the characterization and differentiation of gynecological pelvic masses, using histopathology as the gold standard wherever available.

Materials and methods: This observational study included 60 patients with gynecological pelvic masses detected clinically or on ultrasonography who subsequently underwent MRI pelvis. MRI sequences included T1-weighted, T2-weighted, diffusion-weighted, and contrast-enhanced imaging. Lesions were assessed for site of origin, morphology, internal characteristics, and extent, and MRI findings were correlated with histopathological diagnoses.

Results: Benign lesions constituted the majority of cases. Leiomyoma was the most common pathology, observed in 22 patients (36.7%), followed by adenomyosis in 12 patients (20.0%) and ovarian cysts (simple or hemorrhagic) in 11 patients (18.3%). Other benign lesions included dermoid cysts and tubal pathologies such as hydrosalpinx. Malignant lesions were identified in a smaller proportion of patients, with ovarian carcinoma being the most frequent malignancy (four cases, 6.7%), followed by endometrial carcinoma (three cases, 5.0%) and cervical carcinoma (one case, 1.7%). MRI correctly identified all ovarian lesions, including ovarian carcinomas, cysts, and dermoid cysts, as well as all cases of endometrial and cervical carcinoma. Overall, MRI demonstrated a sensitivity of 100%, specificity of 96.2%, and diagnostic accuracy of 96.7% in differentiating benign from malignant lesions.

Conclusion: MRI is a highly accurate and reliable imaging modality for the evaluation of gynecological pelvic masses, offering excellent lesion characterization and differentiation, thereby significantly aiding clinical decision-making and preoperative planning.

## Introduction

Gynecological malignancies are a worrisome concern for females, among which uterine and ovarian pathologies constitute the major proportion amounting to 30% to 70% overall [1-3], with cervical carcinoma constituting 6.24% prevalence [4].

Among the ovarian pathologies, malignant neoplasms, borderline tumors, and benign cysts have a rising incidence, where imaging holds the key for appropriate distinction between benign and malignant regions [5]. The same applies to uterine pathologies, where fibroids, endometrial carcinomas, and tubal pathologies constitute the majority, and imaging plays a crucial role in identifying and differentiating benign from malignant endometrial invasion, lymphovascular invasion, and nodal status for appropriate management [6].

Compared to conventional ultrasonography, which can identify the lesion in the site, magnetic resonance imaging (MRI) has revolutionized the distinction of gynecological mass lesions (benign vs malignant) as a non-invasive imaging modality. It provides superior contrast in the soft tissues, with multiplanar capability, which holds the key to evaluating pathologies in the pelvic area [7].

Going to the advancements in MRI, it provides valuable insights into the vascularity of the masses with diffusion weighted imaging and dynamic contrast enhancement, enhancing the diagnostic accuracy of differentiating benign from malignant by evaluating the perfusion patterns, cellularity, and septations [8].

Despite these advancements, histopathology remains the gold standard for definitive diagnosis. However, preoperative MRI evaluation is crucial for treatment planning, staging, and surgical decision-making [5-9]. Importantly, existing literature predominantly focuses on individual pathologies or diagnostic accuracy [5-9], with limited studies providing a comprehensive spectrum-based MRI characterization of gynecological masses along with correlation to histopathological outcomes.

Thus, a clear gap exists in systematically describing the MRI spectrum of gynecological lesions and evaluating its diagnostic performance in correlation with histopathology. Addressing this gap may improve preoperative diagnostic confidence and guide clinical decision-making.

Thus, the present study was conducted to comprehensively analyze the MRI spectrum of gynecological masses so that further evidence and more images can be depicted for individual cancers along with histopathological correlation. The primary aim of the study was to describe the MRI spectrum of gynecological masses across uterine and ovarian pathologies. The secondary objectives were to evaluate the diagnostic performance of MRI in differentiating benign and malignant lesions using histopathology as the gold standard, to assess the correlation between MRI findings and histopathological diagnosis, and to identify key MRI features associated with malignancy.

## Materials and methods

An observational cohort study was conducted at Santosh Medical College and Hospital over a period of 18 months during 2024-2025. The study included 60 female patients in whom gynecological pelvic masses were identified on ultrasonography and who were referred for further evaluation with MRI.

Pregnant patients, patients with pelvic masses of non-gynecological origin, those with contraindications to MRI such as pacemakers or ferromagnetic implants (particularly if recently implanted or located within the vicinity of the field of view), patients unwilling to provide written informed consent, and patients without available histopathological confirmation were excluded.

Written informed consent was obtained from all participants prior to enrollment, and the study was carried out after approval from the Institutional Ethics Committee (approval SU/R/2024/1350 (120)), in accordance with ethical principles and the Declaration of Helsinki.

Demographic and clinical details, including age, body mass index, and presenting symptoms, were recorded for all patients. MRI of the pelvis was performed by a 1.5 Tesla United Imaging MR scan (United Imaging Healthcare, Houston, TX, USA), using standard imaging protocols (Table 1). MRI interpretations were performed independently by a radiologist who was blinded to the histopathological outcomes at the time of image analysis to minimize observer bias.

**Table 1:** MRI Protocol parameters for evaluation of gynecological pelvic masses (1.5 Tesla) T2W Fat Suppressed – T2-Weighted Fat-Suppressed Sequence T1W – T1-Weighted Sequence T2W – T2-Weighted Sequence STIR – Short Tau Inversion Recovery DWI (b 50, 800, 1000) – Diffusion-Weighted Imaging (b-values: 50, 800, and 1000 s/mm²) GRE – Gradient Recalled Echo STIR (T2 SPAIR) – Short Tau Inversion Recovery (T2 Spectral Attenuated Inversion Recovery) TR – Repetition Time TE – Echo Time FOV – Field of View

Sequence	Plane	TR (ms)	TE (ms)	Flip Angle (°)	FOV (cm)
T2W Fat Suppressed	Sagittal	4000–6000	80–100	90	22–26
T1W	Sagittal	500–700	10–15	90	22–26
T1W	Axial	500–700	10–15	90	22–26
T2W	Axial	4000–6000	80–100	90	22–26
STIR	Axial	4000–5000	50–70	150	22–26
DWI (b 50, 800, 1000)	Axial	3000–4500	Minimum	90	22–26
GRE	Axial	500–700	15–25	20–30	22–26
T2W	Coronal	4000–6000	80–100	90	22–26
STIR (T2 SPAIR)	Coronal	4000–5000	50–70	150	22–26

Lesions were evaluated for their location, morphology, signal characteristics, and enhancement patterns, and were categorized as benign or malignant based on MRI findings. Histopathological examination of surgically excised specimens was considered the gold standard for final diagnosis. MRI findings were associated with histopathological results to assess diagnostic concordance.

The primary outcomes of the study were MRI spectrum of gynecological pelvic masses and its categorization into benign or malignant using histopathology as the reference standard. Secondary outcomes were sensitivity and specificity for identifying malignant pelvic masses in females.

Statistical analysis

Data were analyzed using Microsoft Excel (Microsoft, Redmond, WA, USA) advanced data software, with categorical variables expressed as frequencies and percentages. The diagnostic performance of MRI in differentiating benign and malignant pelvic masses was assessed by calculating sensitivity, specificity, positive predictive value, negative predictive value, and overall diagnostic accuracy. Sensitivity was defined as the proportion of histopathologically proven malignant lesions correctly identified as malignant on MRI, while specificity was defined as the proportion of histopathologically proven benign lesions correctly identified as benign on MRI. These parameters were calculated using standard 2 × 2 contingency tables.

## Results

The mean age of the patients was 42.1 ± 15.6 years, with an age range of 18 to 85 years. The majority of patients were Hindu (73.3%), followed by Muslims (20.0%), Christians (5.0%), and others including Sikh and Jain (1.7%). Most patients were married (86.7%), while 13.3% were unmarried. With regard to clinical presentation, chronic pelvic pain was the most common symptom, reported by 26.7% of patients. This was followed by menstrual irregularities in 20.0% and postmenopausal bleeding or fever in 16.7% of cases. Other presentations included lower abdominal mass or distension, acute abdominal pain, vaginal bleeding or discharge, and a small proportion of patients who were asymptomatic and detected during routine check-up. Table 2 summarises the demographic characteristics and clinical presentation of the study population.

**Table 2:** Demographic and clinical characteristics of the study population (N = 60)

Category / Parameter	Value
Age (years)	
Mean ± SD age (range)	42.1 ± 15.6 (18–85)
Religion	
Hindu	44 (73.3%)
Muslim	12 (20.0%)
Christian	3 (5.0%)
Others (Sikh/Jain)	1 (1.7%)
Marital Status	
Married	52 (86.7%)
Unmarried	8 (13.3%)
Clinical Presentation	
Chronic pelvic pain	16 (26.7%)
Menstrual irregularities (menorrhagia/menses)	12 (20.0%)
Postmenopausal bleeding / fever	10 (16.7%)
Lower abdominal mass / distension	8 (13.3%)
Acute abdominal pain	6 (10.0%)
Vaginal bleeding / discharge	5 (8.3%)
Asymptomatic / routine check-up	3 (5.0%)

Table 3 shows the distribution of lesions based on pathological diagnosis among the 60 patients studied. Benign lesions constituted the majority of cases. Leiomyoma (fibroids) was the most common benign pathology, observed in 22 patients (36.7%), followed by adenomyosis in 12 patients (20.0%) and ovarian cysts (simple or haemorrhagic) in 11 patients (18.3%). Other benign lesions included dermoid cysts and hydrosalpinx or tubal pathology.

**Table 3:** Distribution of lesions by type (N = 60)

Type of Lesion / Pathological Diagnosis	Frequency (N)	Percentage (%)
Benign lesions (n = 52)		
Leiomyoma (Fibroids)	22	36.7
Adenomyosis	12	20
Ovarian cyst (Simple/Haemorrhagic)	11	18.3
Dermoid cyst (Mature teratoma)	5	8.3
Hydrosalpinx / Tubal pathology / Pyometra	2	3.3
Malignant lesions (n = 8)		
Ovarian carcinoma	4	6.7
Endometrial carcinoma	3	5
Cervical carcinoma	1	1.7
Total	60	100

Malignant lesions were identified in a smaller proportion of patients. Ovarian carcinoma was the most frequent malignant diagnosis, seen in four patients (6.7%), followed by endometrial carcinoma in three patients (5.0%) and cervical carcinoma in one patient (1.7%).

Table 4 shows the distribution between radiological diagnosis and final histopathological findings. Among the 50 cases that were radiologically diagnosed as benign, all were confirmed to be benign on histopathological examination. Of the 10 cases that were reported as malignant or suspicious on radiology, eight were confirmed to be malignant on histopathology, while two were found to be benign. Overall, histopathology identified 52 benign lesions and eight malignant lesions out of the total 60 cases. This indicates that radiology showed good concordance with histopathological diagnosis, particularly in identifying benign lesions. 

**Table 4:** Comparison of radiological and pathological diagnosis (benign vs. malignant)

Radiological Diagnosis	Pathologically Benign	Pathologically Malignant	Total
Benign	50	0	50
Malignant / Suspicious	2	8	10
Total	52	8	60

Overall, MRI demonstrated a sensitivity of 100% and a specificity of 96.2% for the detection of malignant lesions when compared with histopathology. The positive and negative predictive values were 80.0% and 100%, respectively, with an overall diagnostic accuracy of 96.7%.

## Discussion

The present study is significant in delineating the MRI spectrum of gynecological pelvic masses and in differentiating benign from malignant lesions, demonstrating a sensitivity of 100%, specificity of 96.2%, and an overall diagnostic accuracy of 96.7%. However, given the wide and heterogeneous spectrum of gynecological pelvic lesions encountered, these aggregate diagnostic values alone may not fully reflect the clinical utility of MRI. Therefore, individual lesions have been discussed in relation to their organ of origin to provide a more comprehensive and clinically meaningful overview.

Fibroids

Ultrasonography is commonly used as the initial imaging modality due to its cost-effectiveness, portability, real-time assessment, and ability to provide satisfactory anatomical details without radiation exposure. However, MRI remains the most accurate and preferred modality for preoperative evaluation and treatment planning [7]. In the present study, we had 22 cases of leiomyomas (fibroids) and MRI demonstrated high sensitivity and specificity in accurately assessing the number, size, and location of fibroids. It was also particularly effective in identifying fatty, cystic, and hemorrhagic degeneration within fibroids. These findings are consistent with the observations of Schwartz et al. [10], who reported that MRI is superior in differentiating non-cellular variants of leiomyomas, including cystic, fatty, and hemorrhagic degeneration, from typical leiomyomas. However, in the present study, a single case with multiple leiomyomas with tubo-ovarian complex (Ovarian-Adnexal Reporting and Data System (O-RADS) 3 mass) was designated suspicious for malignancy on MRI, which later proved to be benign on histopathology - thereby reinforcing the role of histopathology in suspicious for malignant cases on MRI.

Adenomyosis

In the present study, adenomyosis was identified in 12 cases, highlighting the important role of MRI in its diagnosis. In patients presenting with symptoms such as dysmenorrhea and menorrhagia, MRI demonstrated characteristic findings of diffuse or focal thickening of the junctional zone measuring more than 12 mm on T2-weighted images, which helps in differentiating adenomyosis from uterine fibroids. The presence of small T1 hyperintense cystic foci within the myometrium, representing ectopic endometrial glands, further supported the diagnosis. MRI accurately identified adenomyosis in all cases, with findings correlating well with histopathological results. Similar to the observations reported by Taran et al. [11], adenomyosis was found to coexist with other uterine pathologies in our study, with one patient showing associated fundal uterine fibroid. MRI was effective in clearly distinguishing coexisting lesions, a finding consistent with the study by Togashi et al. [12], which emphasized the utility of MRI in differentiating adenomyosis from leiomyoma, particularly in cases of an enlarged uterus.

Adnexal masses

MRI proved to be the modality of choice for evaluation of adnexal masses in our study due to its high diagnostic accuracy in identifying the organ of origin and in characterizing lesion components such as fat, hemorrhage, solid tissue, and cystic content, thereby facilitating appropriate preoperative planning [13].

In the present study, dermoid cysts were accurately diagnosed on MRI based on characteristic features such as fat-fluid levels, signal suppression on T1 fat-saturated sequences, and the presence of calcifications. These findings are consistent with those described by Togashi et al. [12], who reported that fat within dermoid cysts demonstrates signal intensity similar to subcutaneous fat, suppression on fat-suppressed images, and chemical shift artifacts. Additional features, including internal debris, dermoid plugs, fat-fluid levels, and globular calcifications, further aided confident diagnosis.

Three cases of serous cystadenocarcinoma in our study occurred in patients in the sixth and seventh decades of life, in concordance with findings reported by Salman et al. [5]. MRI reliably differentiated serous from mucinous cystadenomas. Serous cystadenomas appeared as thin-walled unilocular or multilocular cystic lesions with homogeneous T1 hypointensity and T2 hyperintensity, whereas mucinous cystadenomas were larger and multilocular with variable signal intensities reflecting differing mucin content. Benign cystadenomas lacked enhancing solid components, papillary projections, or thick septations, allowing clear distinction from cystadenocarcinomas. Features suggestive of malignancy included large solid components with necrosis, thick-walled cysts, papillary projections, and associated ascites. These findings are consistent with observations by Salman et al. [5], reinforcing the role of MRI in accurate characterization of adnexal masses. Moreover, one case of Krukenberg tumor was also identified as bilateral metastasis to the ovaries in our study, with the primary site being the stomach (adenocarcinoma), identified on upper gastrointestinal endoscopy and biopsy. The patient had a history of unidentified weight loss since six months.

Carcinoma cervix

**Figure 1:**
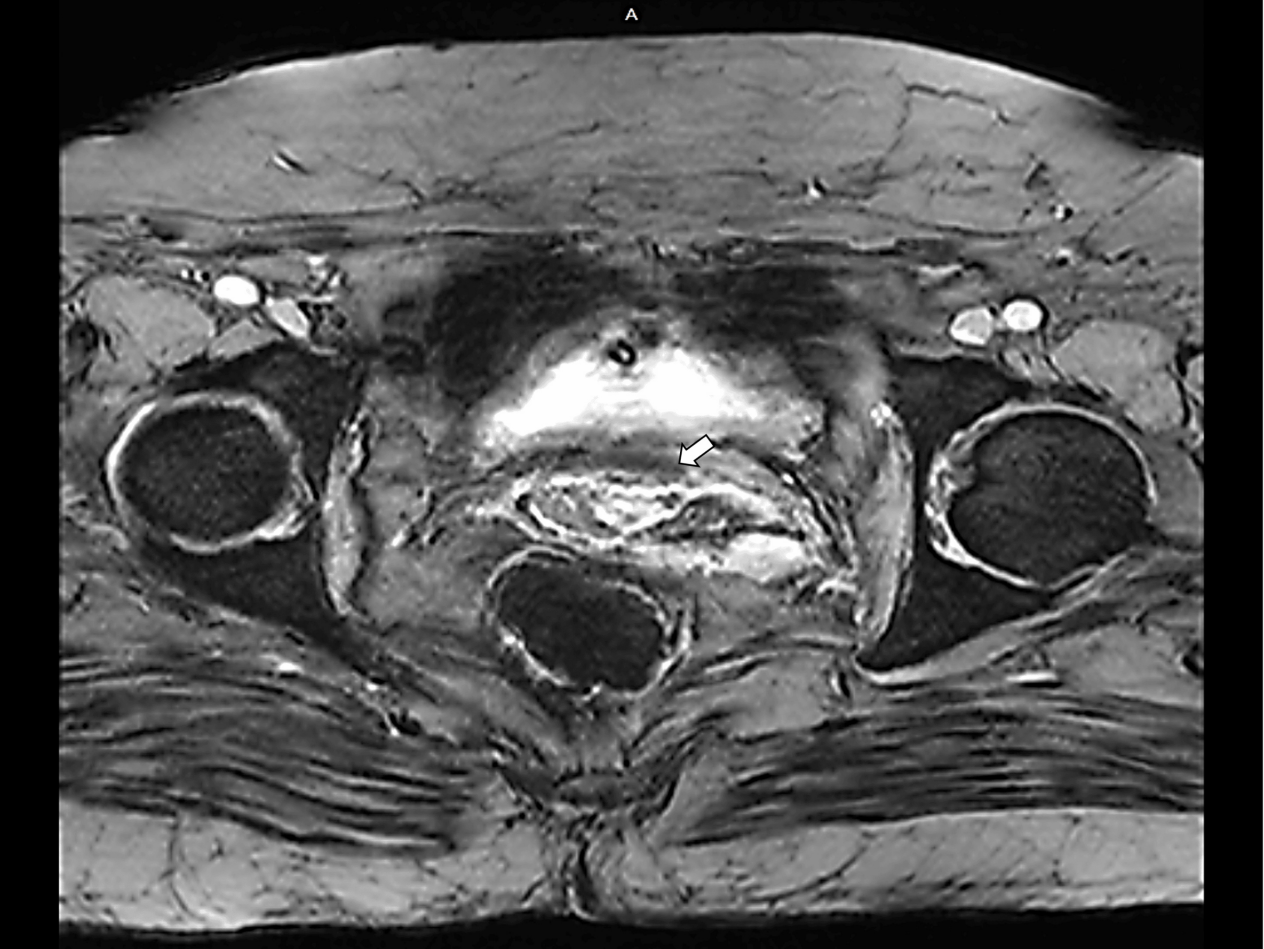
T2-weighted gradient recalled echo (GRE) axial image White arrow showing a well-defined T2 hyperintense lesion in the anterior cervix with preserved fat planes between the lesion and the urinary bladder. No regional lymphadenopathy is seen, and diffuse thickening of the vaginal wall is noted.

**Figure 2:**
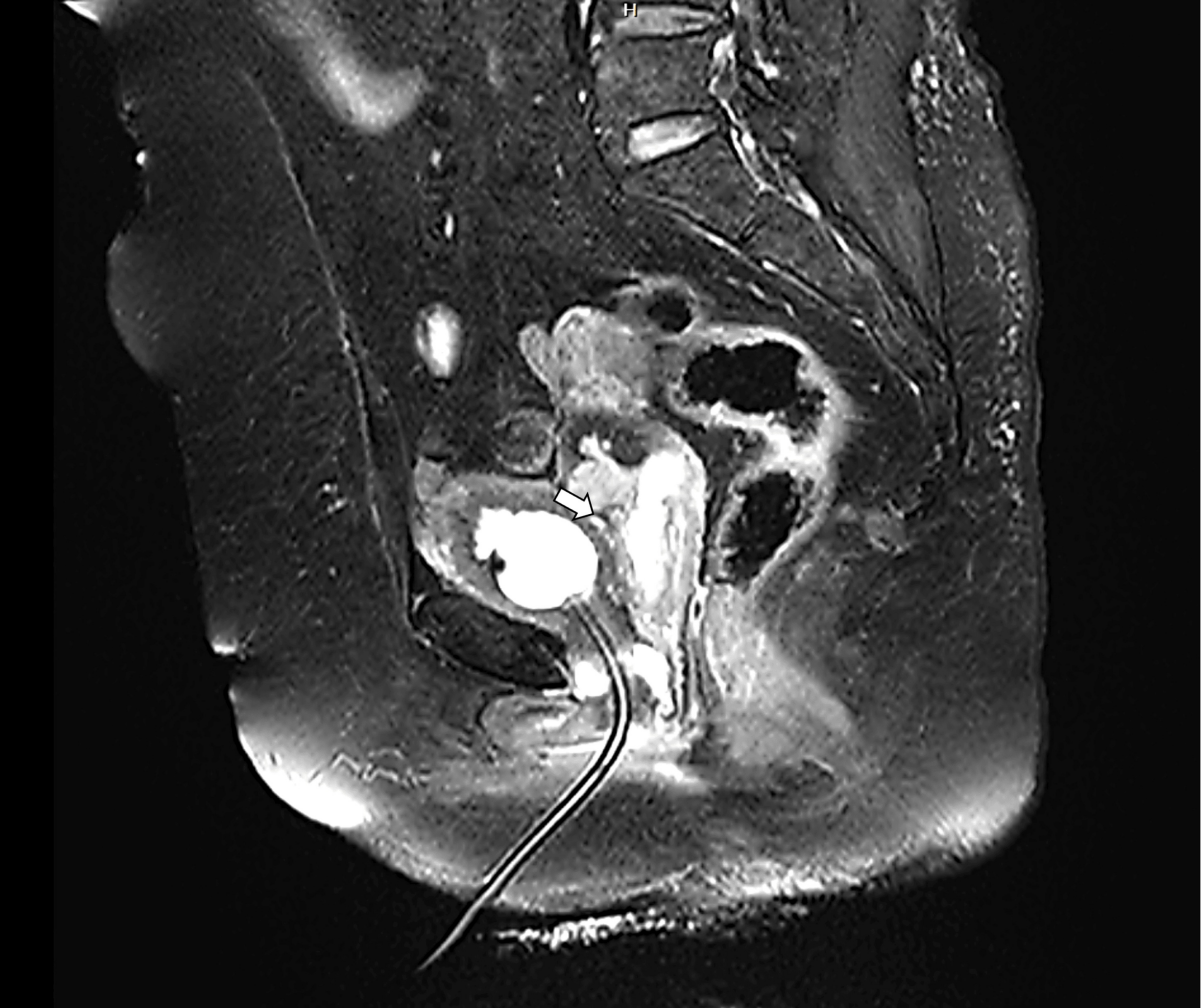
T2-weighted fast spin echo (FSE) sagittal image The arrows shows a T1 hypointense and T2 hyperintense mass localized to the anterior cervix. The uterus is postmenopausal in size with preserved surrounding stromal interfaces.

**Figure 3:**
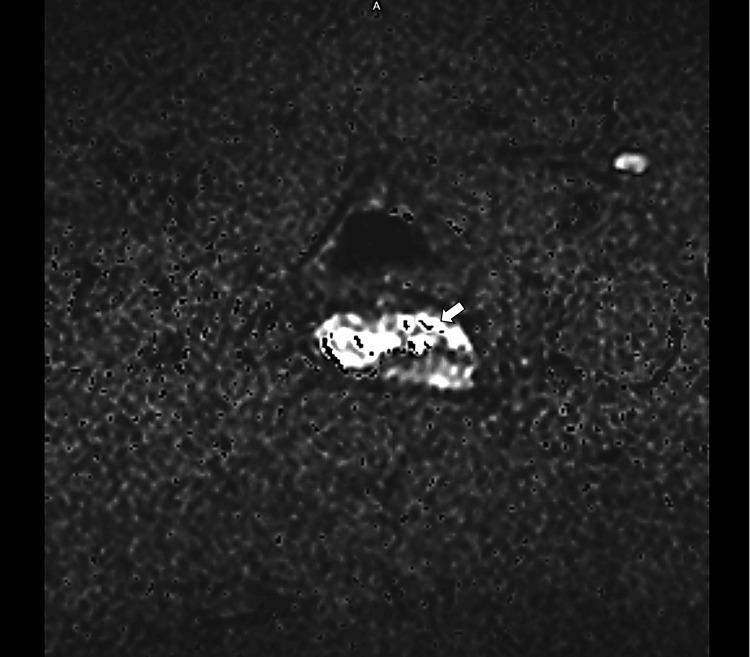
Diffusion-weighted imaging (DWI) with a b-value of 2000 s/mm² demonstrates marked diffusion restriction within the cervical lesion, raising suspicion for malignancy (arrow). b-value – Diffusion sensitivity factor used in diffusion-weighted MRI

**Figure 4:**
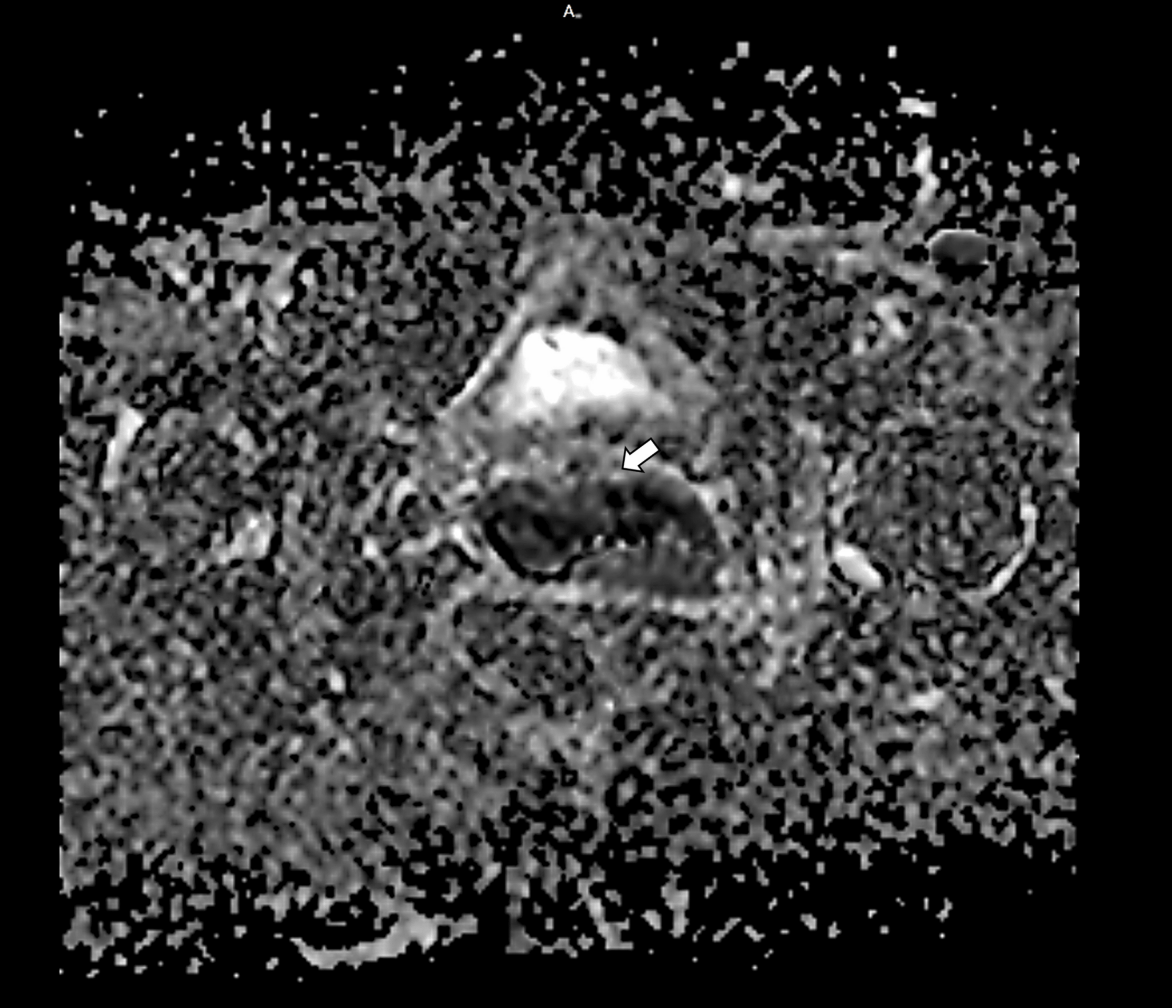
Diffusion-weighted imaging–apparent diffusion coefficient (DWI–ADC) maps show corresponding low ADC values within the lesion (arrow), confirming true restricted diffusion consistent with carcinoma of the cervix.

In the present study, a single case of cervical adenocarcinoma was identified on MRI, although formal staging was not undertaken. MRI enabled confident detection of the lesion based on characteristic imaging features. The cervical lesion appeared as an abnormal mass involving the cervix with intermediate to high signal intensity on T2-weighted images, with disruption of normal cervical stromal architecture. The lesion showed restricted diffusion on diffusion-weighted imaging with corresponding low apparent diffusion coefficient values, supporting its malignant nature. Contrast-enhanced sequences demonstrated heterogeneous enhancement, further aiding in lesion characterization (Figures 1-4).

Although comprehensive staging was beyond the scope of the present study, MRI clearly delineated the primary cervical lesion and its morphology, highlighting its utility in the detection of cervical malignancies. The ability of MRI to provide excellent soft-tissue contrast allows accurate visualization of cervical tumors, particularly endocervical lesions that may be clinically occult, and plays a crucial role in identifying tumor extent, stromal involvement, and adjacent tissue invasion, as emphasized in previous studies [14,15]. These observations reaffirm MRI as the preferred imaging modality for evaluation of carcinoma cervix, even when used primarily for lesion detection rather than staging.

Endometrial carcinoma

**Figure 5:**
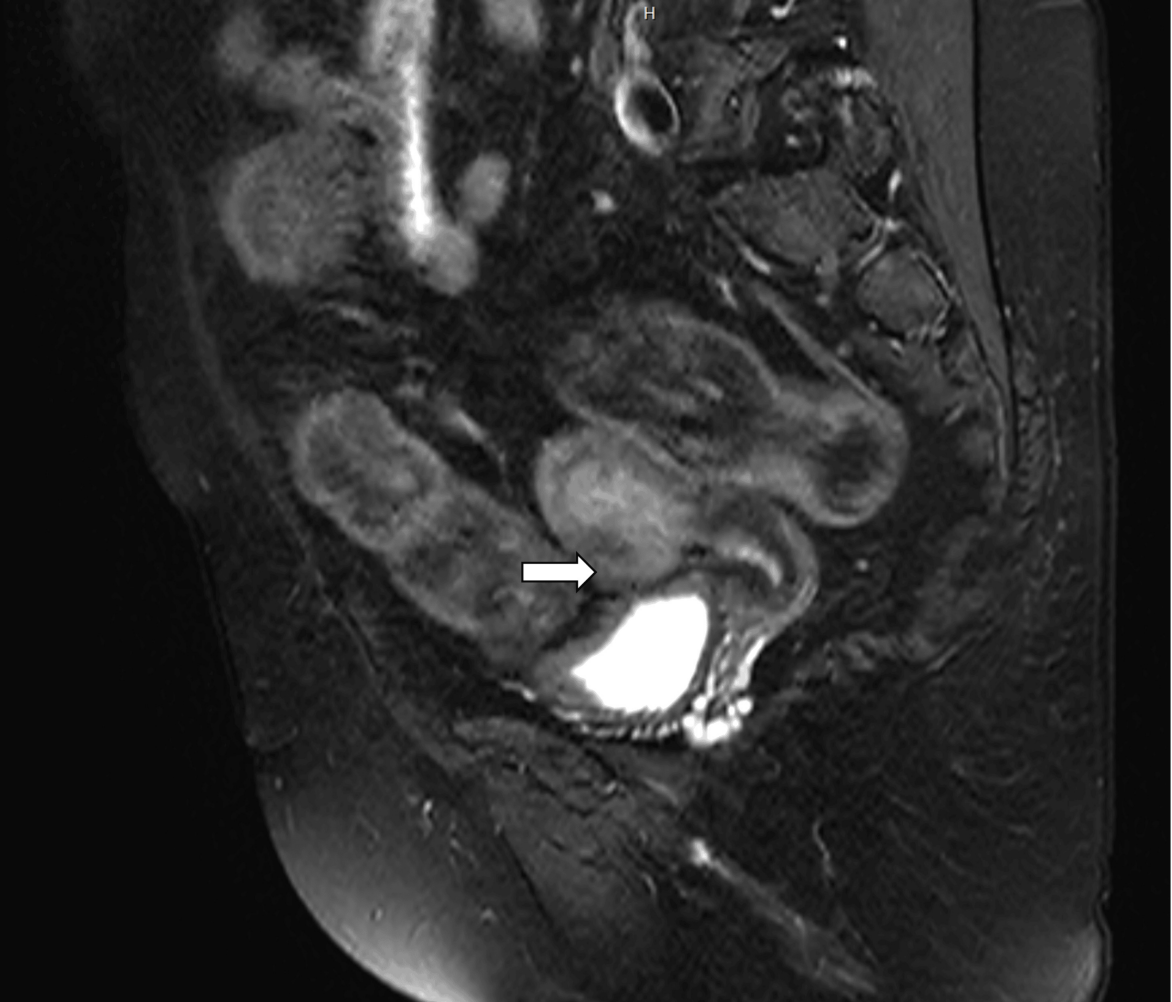
T2-weighted fast spin echo (FSE) SAG image shows an ill-defined, heterogeneously hyperintense endometrial mass (arrow) extending through the full thickness of the myometrium, with suspected serosal involvement. SAG – Sagittal plane

**Figure 6:**
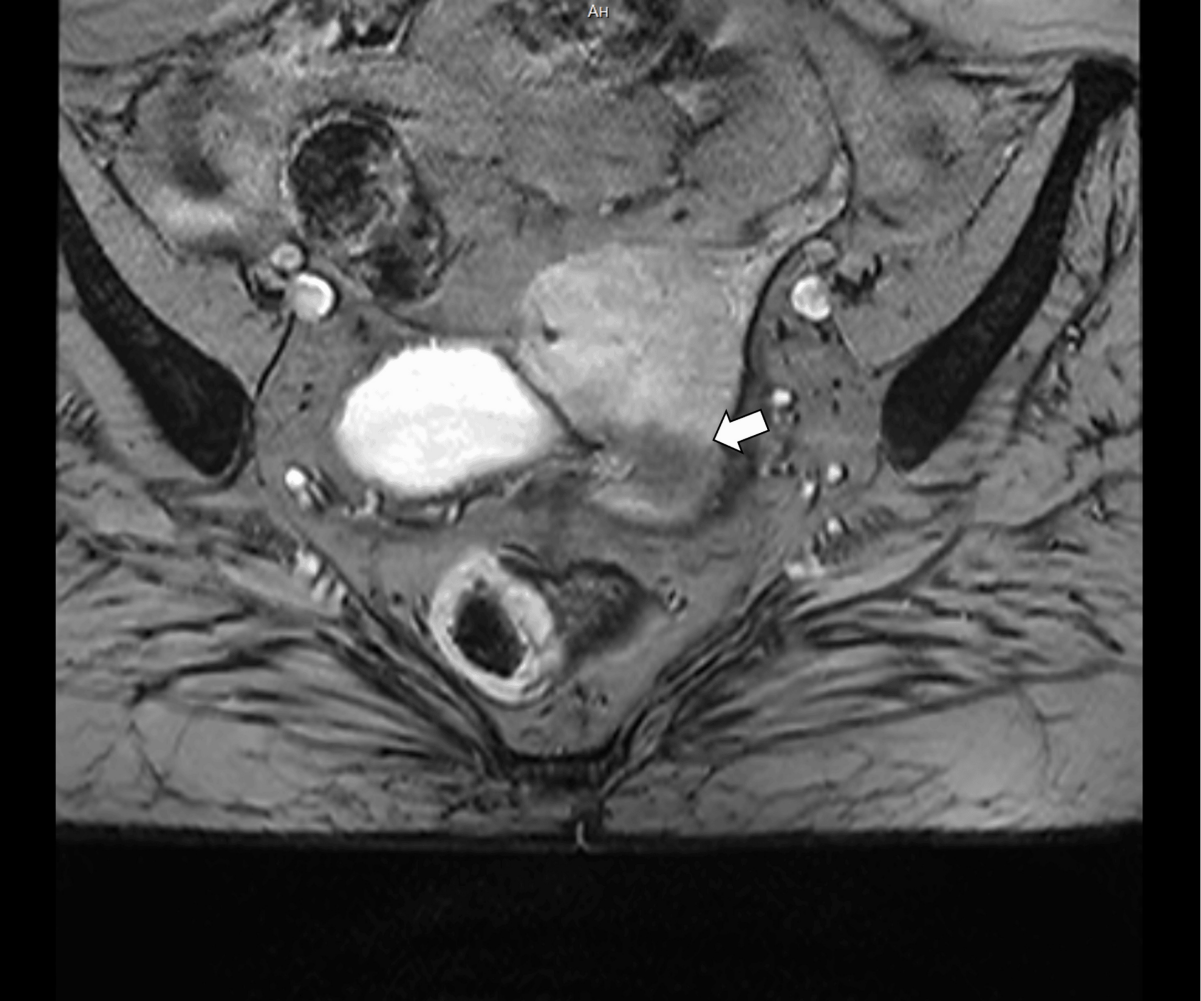
T2-weighted gradient echo (GRE) TRA image showing a heterogeneous mass with areas of intrinsic signal variation (arrows), suggestive of hemorrhagic or necrotic components, with arrows also indicating myometrial invasion. TRA – Transverse (axial) plane

**Figure 7:**
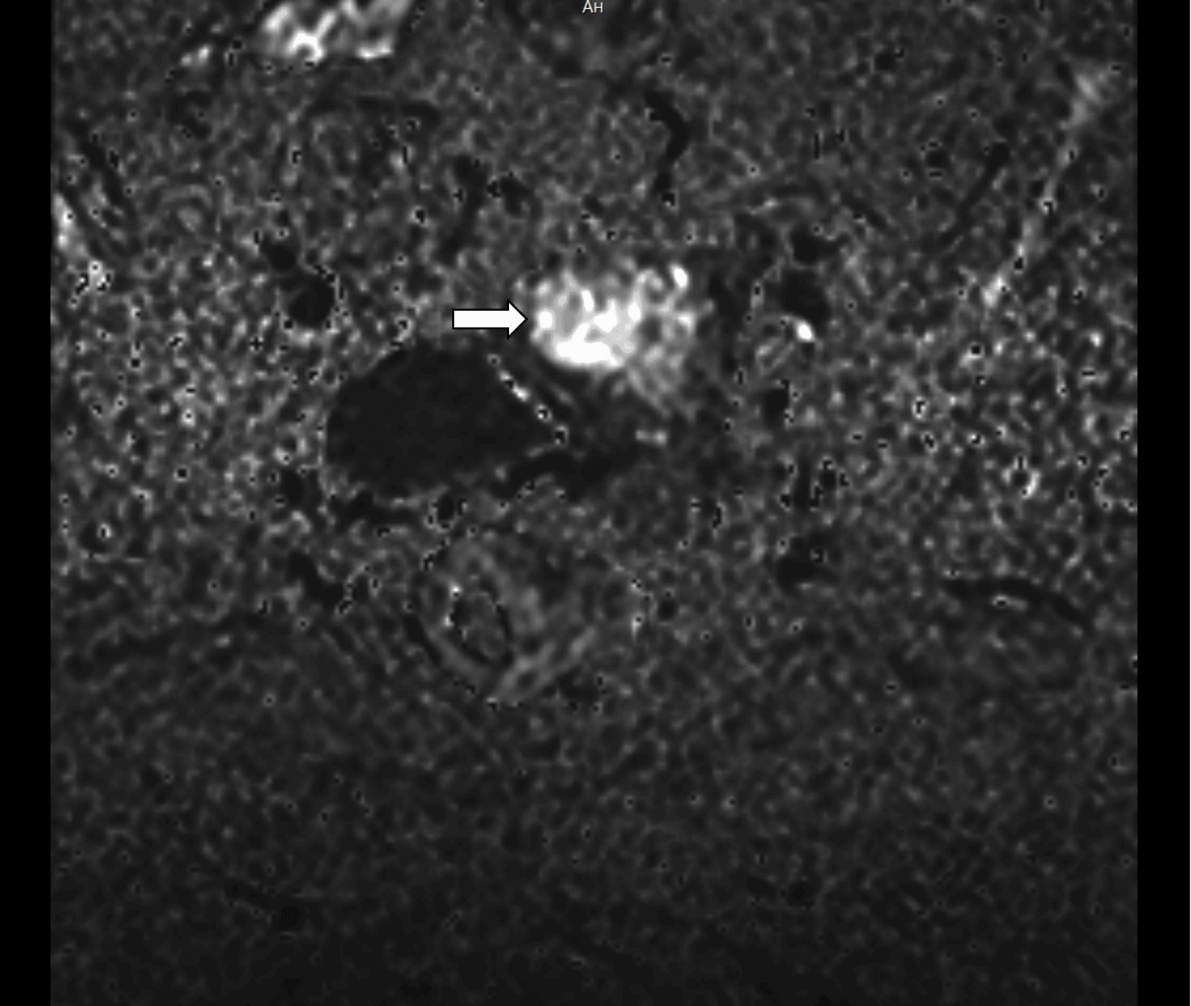
Diffusion-weighted imaging (DWI) with a b-value of 2000 s/mm² demonstrates marked diffusion restriction within the endometrial lesion (arrows), raising suspicion for malignancy. b-value – Diffusion sensitivity factor used in diffusion-weighted MRI

**Figure 8:**
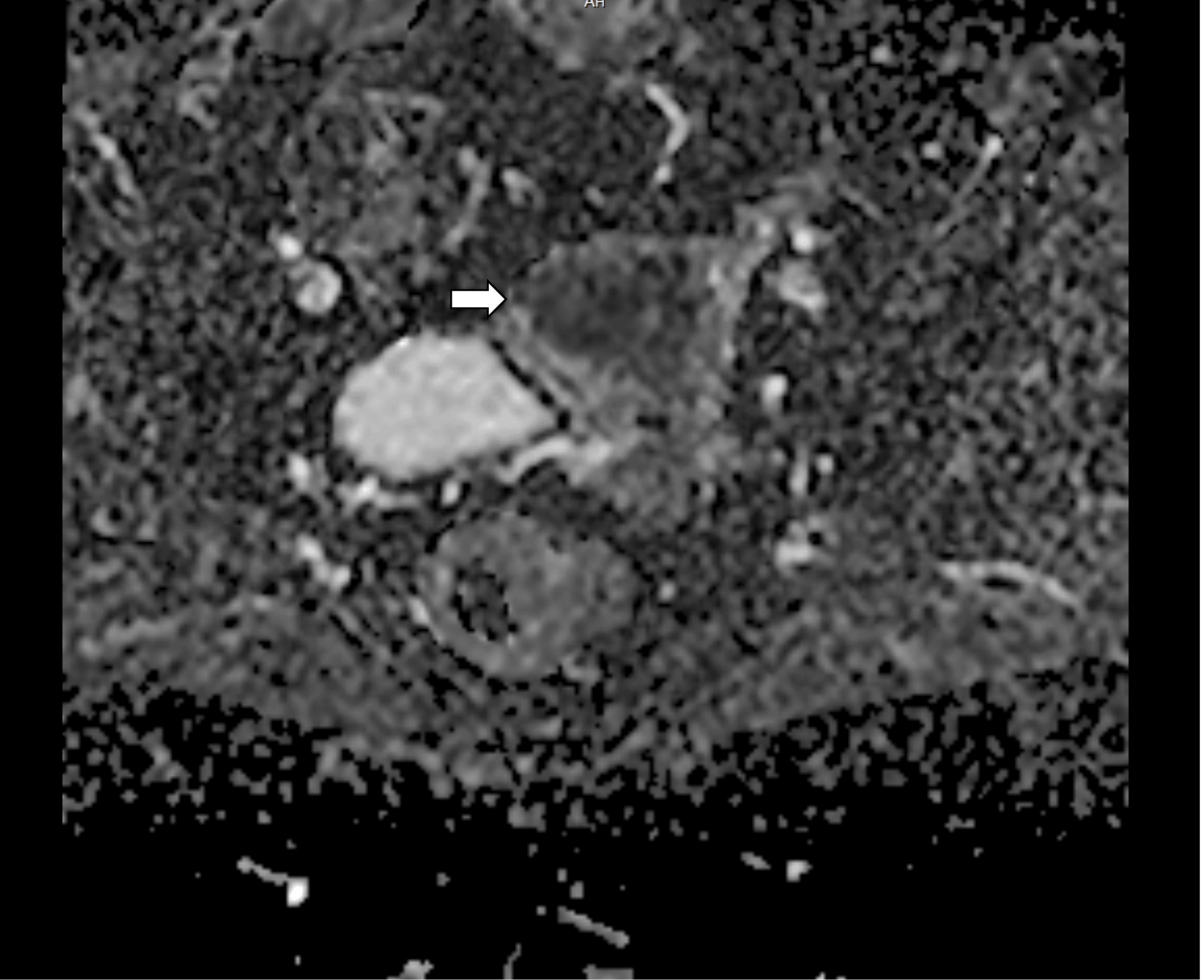
Diffusion-weighted imaging–apparent diffusion coefficient (DWI–ADC) maps show corresponding low ADC values within the lesion (arrow), confirming true restricted diffusion consistent with carcinoma endometrium.

In the present study, three cases of endometrial carcinoma (adenocarcinoma) were correctly identified on MRI. The lesions demonstrated typical imaging features, including abnormal endometrial thickening with intermediate to high signal intensity on T2-weighted images and heterogeneous enhancement on post-contrast sequences. Loss or disruption of the normal low-signal intensity junctional zone suggested myometrial invasion, while diffusion-weighted imaging showed restricted diffusion with corresponding low apparent diffusion coefficient values, supporting the malignant nature of the lesions (Figures 5-8).

Although formal International Federation of Gynecology and Obstetrics (FIGO) staging and assessment of deep myometrial invasion were not undertaken in this study, MRI enabled reliable detection and characterization of endometrial malignancies. The excellent soft-tissue contrast of MRI allows accurate delineation of the endometrial tumor, assessment of myometrial involvement, and evaluation of cervical extension, which are critical for preoperative planning [16].

Limitations of the study

The observational design and single-center setting may limit the generalizability of the findings. The relatively modest sample size may also restrict the evaluation of less common gynecological lesions. The study did not utilize a standardized MRI reporting system such as O-RADS MRI, which may limit comparability with other studies. Interobserver variability was not assessed, as imaging interpretation was performed by a single radiologist. Additionally, advanced statistical measures such as confidence intervals and hypothesis testing were not included due to the limited sample size. Future studies incorporating structured reporting systems, multiple observers, and more robust statistical analyses are warranted.

## Conclusions

MRI proved to be an effective modality for the evaluation and characterization of gynecological pelvic masses in the present study, offering excellent soft-tissue contrast and detailed anatomical delineation for identifying the site of origin and differentiating benign from malignant lesions. Using histopathology as the reference standard, MRI demonstrated high diagnostic performance, with sensitivity of 100%, specificity of 96.2%, and overall accuracy of 96.7%. However, these findings should be interpreted with caution in view of the relatively small sample size, single-center design, and the absence of confidence interval estimation, which may limit the precision and generalizability of the results.

MRI also enabled characterization of a wide spectrum of uterine and adnexal pathologies, including fibroids, adenomyosis, ovarian cysts, and gynecological malignancies, thereby supporting its role in preoperative evaluation. These findings are broadly consistent with existing literature reporting high diagnostic accuracy of MRI in pelvic mass assessment.

Although formal staging was not uniformly performed, MRI provided valuable information regarding lesion extent and internal characteristics such as hemorrhage, fat content, and solid components. Overall, MRI serves as a reliable non-invasive imaging tool in the evaluation of gynecological masses; however, larger studies with standardized protocols and robust statistical analyses, including confidence interval estimation, are required to further validate and generalize these findings.
